# Novel homozygous *OSGEP* gene pathogenic variants in two unrelated patients with Galloway-Mowat syndrome: case report and review of the literature

**DOI:** 10.1186/s12882-019-1317-y

**Published:** 2019-04-11

**Authors:** Andrea Domingo-Gallego, Mónica Furlano, Marc Pybus, Daniel Barraca, Ana Belén Martínez, Emiliano Mora Muñoz, Roser Torra, Elisabet Ars

**Affiliations:** 1 Molecular Biology Laboratory, Fundació Puigvert, Instituto de Investigaciones Biomédicas Sant Pau (IIB-Sant Pau), Universitat Autònoma de Barcelona, REDinREN, Instituto de Investigación Carlos III, Cartagena 340-350, 08025 Barcelona, Catalonia Spain; 2 Nephrology Department, Fundació Puigvert, Instituto de Investigaciones Biomédicas Sant Pau (IIB-Sant Pau), Universitat Autònoma de Barcelona, REDinREN, Instituto de Investigación Carlos III, Barcelona, Catalonia Spain; 3 Hospital General Universitario Gregorio Marañón, Madrid, Spain; 4 Hospital Universitario Mutua de Terrassa, Barcelona, Catalonia Spain

**Keywords:** Galloway-Mowat syndrome, Nephrotic syndrome, *OSGEP*, KEOPS complex, Genetic testing, Case report

## Abstract

**Background:**

Galloway-Mowat syndrome (GAMOS) is a rare autosomal recessive disorder characterized by early-onset nephrotic syndrome and microcephaly with brain anomalies. *WDR73* pathogenic variants were described as the first genetic cause of GAMOS and, very recently, four novel causative genes, *OSGEP, LAGE3*, *TP53RK*, and *TPRKB*, have been identified.

**Case presentation:**

We present the clinical and genetic characteristics of two unrelated infants with clinical suspicion of GAMOS who were born from consanguineous parents. Both patients showed a similar clinical presentation, with early-onset nephrotic syndrome, microcephaly, brain atrophy, developmental delay, axial hypotonia, and early fatality. We identified two novel likely disease-causing variants in the *OSGEP* gene. These two cases, in conjunction with the findings of a literature review, indicate that *OSGEP* pathogenic variants are associated with an earlier onset of nephrotic syndrome and shorter life expectancy than *WDR73* pathogenic variants.

**Conclusions:**

Our findings expand the spectrum of pathogenic variants in the *OSGEP* gene and, taken in conjunction with the results of the literature review, suggest that the *OSGEP* gene should be considered the main known monogenic cause of GAMOS. Early genetic diagnosis of GAMOS is of paramount importance for genetic counseling and family planning.

## Background

Galloway-Mowat syndrome (GAMOS) (OMIM #251300) is a rare autosomal recessive syndrome first described in 1968. It is a clinically heterogeneous condition characterized by early-onset nephrotic syndrome associated with microcephaly, gyral abnormalities of the brain, and delayed psychomotor development. Most patients also present dysmorphic facial features, including hypertelorism, ear abnormalities, and micrognathia. Most affected individuals die in early childhood [1, 2]. The estimated prevalence is < 1/1,000,000 but it is likely that many cases remain misdiagnosed or undiagnosed.

Truncating variants in *WDR73* gene were described as the first monogenic cause of GAMOS in two families [3]. The protein encoded by this gene is implicated in the regulation of the microtubule network during cell cycle progression, proliferation, and survival [3–5]. Homozygous missense variants in the *WDR73* gene were later reported [6, 7]. Recently, pathogenic variants in the *OSGEP, LAGE3*, *TP53RK*, and *TPRKB* genes have been identified as novel monogenic causes of GAMOS. These genes encode four subunits of the evolutionary highly conserved KEOPS (kinase, endopeptidase, and other proteins of small size) complex. This complex plays an important role in brain and renal development*.* To date, the genetic cause of GAMOS has been reported in 54 families: *OSGEP* in 26 (48.15%), *WDR73* in 19 (35.18%), *TP53RK* in 4 (7.40%), *LAGE3* in 3 (5.55%), and *TPRKB* in 2 (3.70%) [2–11].

Here, we report two unrelated patients with GAMOS carrying homozygous pathogenic variants in the newly identified *OSGEP* gene.

## Case presentation

**Fig. 1:**
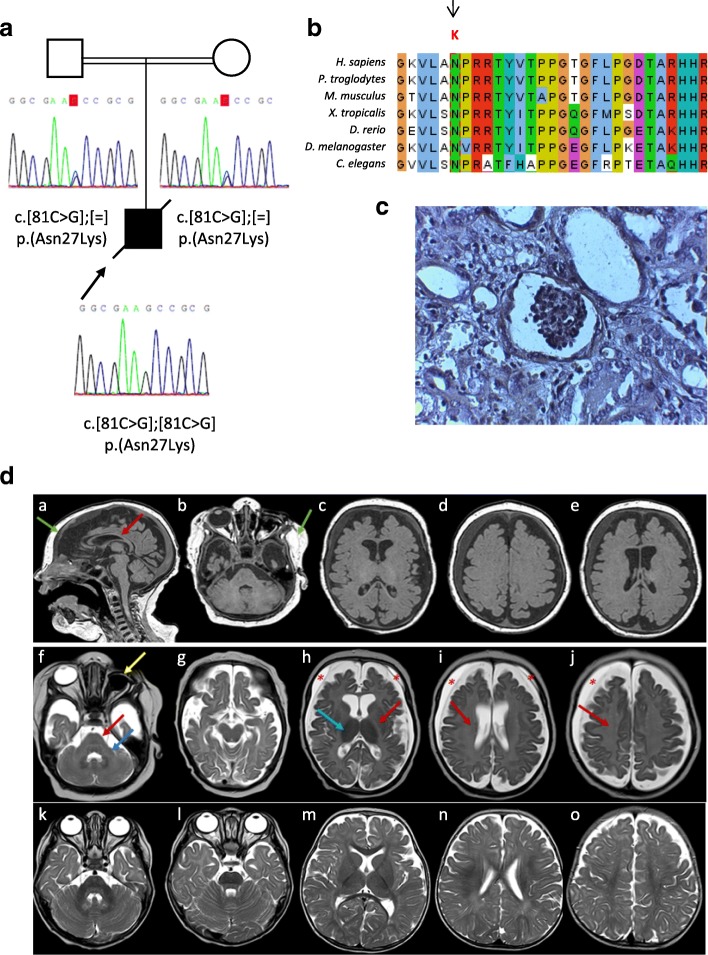
**a** Pedigree of patient 1 with a likely pathogenic *OSGEP* variant, c.81C > G p.(Asn27Lys), in homozygosity while his consanguineous parents are healthy heterozygous carriers. **b** The identified missense variant c.81C > G p.(Asn27Lys) affects a totally conserved amino acid N27 in OSGEP orthologs. **c** Silver-stained renal biopsy from patient 1 showed glomerular collapse with mesangial matrix increase, atrophic tubules, and interstitial fibrosis on light microscopy. **d** CMRI performed in patient 1 at 8 months of age: sagittal 3 Dimensional Imaging T1 sequence (*a*), axial reconstructions (*b*–*e*), and axial Turbo Spin Echo (TSE) T2 (*f*–*j*). *k*–*o*: Sequence TSE T2 of normal control individual. MRI revealed craniofacial disproportion in relation to microcephaly (*a*); supratentorial cortico-subcortical atrophy with increased extra-axial space, prominence of the frontal horns, and thinning of the corpus callosum (red arrow in a); bilateral subdural frontoparietal hygromas (red asterisks in *h*–*j*); and atrophy of the basal ganglia (*h*). A decrease in the number and depth of the grooves was observed (*h*–*j*) and there was an absence of normal myelination of the brainstem (red arrow in *f*), cerebellar peduncles (blue arrow in *f*), internal capsules (red arrow in *h*), and white bihemispheric substance (arrows in *i* and *j*). Hypointense T2 signal of the thalamus was evident (blue arrow in *h*). Enucleation of the left eye is denoted by the yellow arrow in *f*. Finally, there was an increase in the thickness of the cranial and facial subcutaneous cellular tissue (green arrows in *a* and *b*)

Patient 1 was a first-child male born to healthy consanguineous parents from Spain with no previous family history of kidney disease (Table 1, Fig. 1A). He was born at 39.4 weeks of gestation. Birth weight was 3400 g, height was 51 cm, and head circumference was 34 cm, with no dysmorphic features. Forty-five days after birth, the patient was diagnosed as having congenital nephrotic syndrome with severe proteinuria, hypertension, and hypothyroidism. He also presented edema, hyperkalemia, hyponatremia, and hypomagnesemia. Renal ultrasound showed poor corticomedullary differentiation in the right kidney. Renal biopsy showed diffuse mesangial sclerosis, tubular atrophy, and primitive glomeruli (Fig. 1C). The patient progressed to end-stage renal disease and required peritoneal dialysis. He also presented left eye evisceration, dry right eye, and gastroesophageal reflux. At 5 months of age, the neurological examination revealed microcephaly with a head circumference of 38.5 cm (percentile < 1, − 4.72 SD), severe psychomotor delay for his age, and axial hypotonia. Cranial magnetic resonance imaging (CMRI) revealed brain atrophy and absence of normal myelination of the brainstem, cerebellar white matter, bilateral hemispheric white matter, internal capsules, and corpus callosum as well as abnormal intensity signal in the dentate nucleus and thalamus (Fig. 1D). In view of the congenital nephrotic syndrome and microcephaly with brain anomalies, a clinical diagnosis of GAMOS was suspected. The patient presented progressive neurological deterioration and died at 8 months of age.

**Table 1:** Clinical data of patients 1 and 2

Clinical features	Patient 1	Patient 2
Sex	Male	Female
Age at death	8 months	7 months
Origin	Spanish	Pakistani
Neonatal profile (at birth)		
Gestational period (weeks)	39.4	40.3
Weight (g)	3400 (58th percentile)	2940 (17th percentile)
Height (cm)	51 (70th percentile)	49 (30th percentile)
Head circumference (cm)	34 (28th percentile)	32 (3rd percentile)
Renal phenotype		
Onset of NS (days)	45	75
Renal biopsy	DMS, tubular atrophy, primitive glomeruli	Increased glomerular mesangial matrix
Renal ultrasound	Poor corticomedullary differentiation	Cortical hyperechogenicity
Hyperkalemia	Yes	No
Hypomagnesemia	Yes	No
Neurological involvement		
Brain MRI	Microcephaly, brain atrophy, poor myelination	Microcephaly, brain atrophy
Developmental delay	Yes	Yes
Axial hypotonia	Yes	Yes
Skeletal abnormalities	No	Arachnodactyly
Dysmorphic features	No	Yes (wide nasal bridge, retrognathia, low set ears)
Others	Dry right eye Gastroesophageal reflux	Epileptiform activity Seizures

*DMS* Diffuse mesangial sclerosis, *NS* nephrotic syndrome

**Fig. 2:**
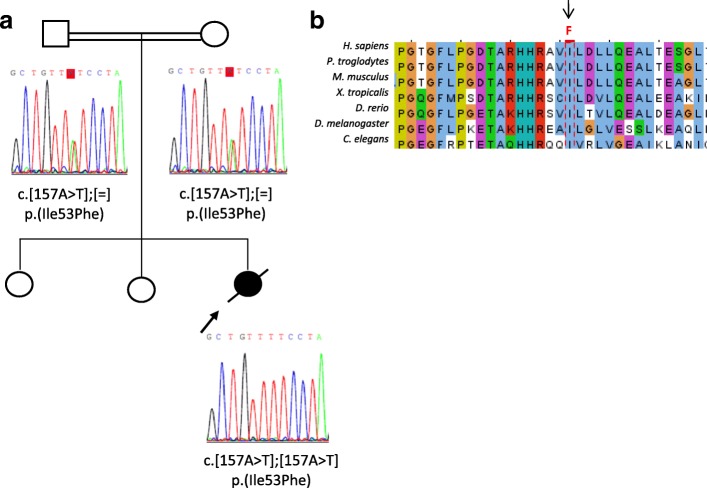
**a** Pedigree of the family of patient 2 with a likely pathogenic *OSGEP* variant c.157A > T p.(Ile53Phe) in homozygosity in the proband (arrow) and heterozygosity in her healthy parents. **b** Conservation of I53 in OSGEP orthologs to *C. elegans*

Patient 2 was a female infant with normal karyotype (46, XX) born to healthy consanguineous parents from Pakistan. The patient had two healthy sisters and there was no family history of kidney disease (Table 1, Fig. 2a). She was born at 40.3 weeks of gestation. Birth weight was 2940 g, height was 49 cm, and head circumference was 32 cm with signs of microcephaly. Screening for metabolic disorders and cerebral ultrasound were normal. The patient presented dysmorphic features (wide nasal bridge, aquiline nose and retrognathia, low set ears, and arachnodactyly) with axial hypotonia and poor eye contact. Seventy-five days after birth, she presented with nephrotic range proteinuria, hypoproteinemia with severe hypoalbuminemia, hypertriglyceridemia and hypercholesterolemia. Serum creatinine and urea were normal. Abdominal ultrasound showed normal-sized kidneys and correct corticomedullary differentiation with cortical hyperechogenicity, bilateral pleural effusion, and a discrete amount of fluid in the abdominal cavity. Renal biopsy showed one glomerulus with increased mesangial matrix and two normal glomeruli; fibrosis and tubular atrophy were absent. CMRI revealed severe brain atrophy with normal cerebellum and brainstem. Electroencephalogram showed normal brain activity with low-amplitude brain waves and occasional frontal left epileptiform activity. The patient evolved with failure to thrive, anemia, and electrolyte disorders and finally died from cardiorespiratory arrest in a sepsis context at 7 months of age.

### Genetic study

Variant analyses of patients 1 and 2 were performed by targeted massive parallel sequencing using an updated version of our kidney disease gene panel that includes more than 200 genes causative of or associated with inherited kidney diseases (including *WDR73*, *TPRKB*, *TP53RK*, *LARGE3*, and *OSGEP* genes) [12].

Briefly, libraries were prepared according to the manufacturer’s standard protocol, NimbleGen SeqCap EZ Library SR version 4.3. Patients’ DNAs were fragmented and hybridized to the custom NimbleGen SeqCap EZ Choice gene panel and sequenced on a NextSeq 500 instrument (Illumina). Sequence data analysis was performed using an open-source in-house bioinformatic pipeline, as previously reported [12–14]. The mean depth of coverage per exon of *OSGEP* ranged from 153 to 433, with 100% of the bases covered at least 100X. Prediction of pathogenicity was evaluated using different bioinformatic algorithms (DANN, GERP, dbNSFP.FATHMM, LRT, MetaLR, MetaSVM, MutationAssessor, PROVEAN, SIFT, and MutationTaster). Clinical interpretation of variants was based on American College of Medical Genetics (ACMG) recommendations [15]. All candidate pathogenic variants were validated by conventional polymerase chain reaction amplification and Sanger sequencing. Familial segregation analysis was assessed. Analysis of copy number variations (CNVs) was performed using CoNVaDING (copy number variation detection in next-generation sequencing gene panels) software [16].

Patient 1 carried a homozygous missense variant c.81C > G p.(Asn27Lys) in exon 1 of the *OSGEP* gene (NM_017807), not previously described in the literature. This variant was predicted to be pathogenic by seven prediction tools (DANN, GERP, LRT, MutationAssessor, MutationTaster, SIFT, and PROVEAN) and benign by three (dbNSFP.FATHMM, MetaLR, and MetaSVM). This variant altered an evolutionarily highly conserved residue and was absent from the population databases Genome Aggregation Database (gnomAD) and 1000 Genomes (Fig. 1B). Segregation analysis showed that both parents were heterozygous carriers of this *OSGEP* variant (Fig. 1A). We concluded that this variant was likely pathogenic (Table 2).

**Table 2:** Information of the two identified variants in the *OSGEP* gene

		Population Databases			Predictor scores											
Patient	Variant	dbSNP	1000G	gnomAD ƒ	DANN	GERP	LRT	MA	MT	SIFT	PROVEAN	dbNSFP.FATHMM	MetaLR	MetaSVM	Parental segregation confirmed	ACMG (Variant classification)
Patient 1	c.81C > G p.(Asn27Lys)	No	0	0	P	P	P	P	P	P	P	B	B	B	Yes	LP (PM2, PM3, PP1, PP2, PP3, PP4)
Patient 2	c.157A > T p.(Ile53Phe)	rs780944919	0	0.0000979 (South Asians)	P	B	P	P	P	B	P	B	B	B	Yes	LP (PM2, PM3, PP1, PP2, PP4)

*1000G* 1000 Genomes Project, *ƒ* Allele Frequency, *B* Benign, *gnomAD* genome Aggregation Database, *LP* Likely Pathogenic, *MA* MutationAssessor, *MT* MutationTaster, *P* Pathogenic

Patient 2 carried a homozygous missense variant c.157A > T p.(Ile53Phe) localized in exon 2 of the *OSGEP* gene. This variant has not been previously reported in literature. The variant c.157A > T p.(Ile53Phe) was predicted to be pathogenic by five prediction tools (DANN, LRT, MutationAssessor, MutationTaster, and PROVEAN) and benign by five (GERP, dbNSFP.FATHMM, MetaLR, SIFT, and MetaSVM). This variant was conserved in *OSGEP* orthologs to *C. elegans* and is extremely rare in the general population (Fig. 2b), with a minor allele frequency in South Asians is 0.00009799 (3 of 30,782 sequenced alleles, no homozygous individuals) in the gnomAD database. The global allele frequency was lower than the 0.0001 threshold for recessive gene *OSGEP*. The parents were confirmed to be heterozygous carriers (Fig. 2a). We classified this variant as likely pathogenic (Table 2).

## Discussion and conclusions

We report two patients who presented with nephrotic syndrome with onset at < 3 months old, primary microcephaly, and developmental delay, which are hallmarks of GAMOS. Both patients carried homozygous likely disease-causing variants in the *OSGEP* gene. This gene was recently identified as causative of GAMOS in a large cohort of 907 individuals with nephrotic syndrome [2]. Pathogenic variants in one of the four genes *TP53RK*, *TPRKB*, *LAGE3*, and *OSGEP*, encoding KEOPS complex subunits, were found in 37 out of 91 patients with GAMOS. Independently, a homozygous pathogenic variant in the *OSGEP* gene was reported in two siblings with a similar renal-neurological phenotype, also by whole exome sequencing [8].

The *OSGEP* gene encodes the *O*-sialoglycoprotein endopeptidase enzyme, which regulates the second biosynthetic step in the formation of *N*-6-threonylcarbamoyladenosine in the cytosol, essential for mRNA translational initiation and efficiency. The highly conserved KEOPS complex is implicated in several cell processes, such as control of telomere length, telomere-associated DNA damage response signaling, and genome maintenance. Zebrafish larvae knockout of the *osgep* gene resulted in primary microcephaly, with increased apoptosis in the brain compared with controls and early lethality. Knockout mouse embryos also showed microcephaly compared with wild-type embryos. Neither mutant fish nor mice showed any renal phenotype, possibly due to embryonic early lethality [2].

Great strides have been made in the understanding of GAMOS disease over the past 4 years, with the identification of its genetic bases in some patients. However, the genetic etiology of more than three-quarters of patients with a clinical diagnosis of GAMOS remains elusive, suggesting that additional causative genes remain to be identified. Currently, the principal known causative genes of GAMOS are *OSGEP* and *WDR73*.

A review of the literature based on 31 patients (26 families) bearing *OSGEP* pathogenic variants and 23 patients (13 families) with *WDR73* pathogenic variants indicates that *OSGEP* causes earlier onset of nephrotic syndrome than *WDR73* [2–4, 6–10]. Eighty percent (25/31) of patients with *OSGEP* pathogenic variants developed nephrotic syndrome with a mean age at onset of 10.36 months (ranging onset from birth to 13 years). In comparison, 35% (8/23) of patients with *WDR73* pathogenic variants presented nephrotic syndrome at a mean age of 7.7 years (ranging from 0.5 to 16 years). Our two patients carrying *OSGEP* pathogenic variants presented with nephrotic syndrome before 3 months of age.

Renal manifestations described in GAMOS patients vary from isolated proteinuria to steroid-resistant nephrotic syndrome, and some patients even have no renal alterations during follow-up period [2–8]. Intrafamilial clinical variability has also been described in GAMOS. For instance, two siblings carrying a *WDR73* pathogenic variant manifested contrasting renal phenotype [3]. One of the affected siblings presented with nephrotic syndrome at the age of 5 years, rapidly developed chronic renal insufficiency, and died after a month, while the other had no renal symptoms at the age of 7 years [3]. A homozygous *OSGEP* pathogenic variant, c.974A > G p.(Arg325Gln), has also been associated with renal tubular anomalies [10]. It was detected in a girl with magnesium-wasting tubulopathy and partial Fanconi syndrome with a normal glomerular filtration rate who never developed nephrotic syndrome [10]. Interestingly, this variant was previously identified in two siblings with severe hypomagnesemia, hypercalciuria, and proteinuria but normal albumin levels [8]. The authors raised the question of whether these patients should be considered to be affected by a different clinical entity [8, 10].

The review of the literature also indicates that patients with *OSGEP* pathogenic variants have a shorter life expectancy than those with *WDR73* pathogenic variants. Seventy-one percent (22/31) of patients with *OSGEP* pathogenic variants died at a mean age of 1.5 years (ranging from 6 weeks to 8 years). In line with these reported cases, our patients died at 8 and 7 months of age. However, seven patients with *OSGEP* pathogenic variants were alive at 13 (2), 10.5 (1), 7 (1), 3.5 (1), and 2 (1) years and at 7 (1) months [2, 10]. It should be noted that four of them carried the above-mentioned *OSGEP* variant, c.974A > G p.(Arg325Gln), associated with renal tubular anomalies [10]. Twenty-two percent (5/23) of patients carrying pathogenic variants in the *WDR73* gene died at a mean age of 8.1 years (ranging from 2.5 to 17 years).

Nearly all *OSGEP* variants reported as causative of GAMOS are missense, except for two splicing variants [2, 8]. These variants are located throughout the *OSGEP* gene. By contrast, different types of variant in *WDR73* causative of GAMOS have been reported in the literature, including nonsense (3), frameshift (3), and missense (4). No correlation seems to exist between the type or position of the variant and particular clinical features. Identification of the causative pathogenic variant in patients 1 and 2 confirmed the initial clinical suspicion of GAMOS and allowed precise genetic counseling to their parents. In particular, it allowed prenatal diagnosis of a baby girl without GAMOS for the parents of patient 1.

In conclusion, we report two patients with GAMOS caused by *OSGEP* pathogenic variants. These two cases, in conjunction with the reported cases in the literature, add evidence that *OSGEP* pathogenic variants are the most prevalent cause of GAMOS and are associated with a more severe phenotype than *WDR73* pathogenic variants. For these reasons, *OSGEP* variant analysis should be considered as the first step in genetic diagnosis of patients with clinical suspicion of GAMOS; this is especially true for those labs that do not perform massive parallel sequencing and for those cases with early and severe onset of the disease. Genetic diagnosis of GAMOS is of paramount importance for genetic counseling and family planning and allows prenatal or preimplantation genetic diagnosis for future pregnancies.

## Abbreviations

CMRI: Cranial magnetic resonance imaging
DMS: Diffuse mesangial sclerosis
GAMOS: Galloway-Mowat syndrome
gnomAD: Genome Aggregation Database
KEOPS: Kinase, endopeptidase and other proteins of small size
TSE: Turbo Spin Echo
